# Creating highly efficient resistance against wheat dwarf virus in barley by employing CRISPR/Cas9 system

**DOI:** 10.1111/pbi.13077

**Published:** 2019-02-05

**Authors:** András Kis, Éva Hamar, Gergely Tholt, Rita Bán, Zoltán Havelda

**Affiliations:** ^1^ National Agricultural Research and Innovation Centre Agricultural Biotechnology Institute Gödöllő Hungary; ^2^ Georgikon Faculty Festetics Doctoral School University of Pannonia Keszthely Hungary; ^3^ Plant Protection Institute Centre for Agricultural Research Hungarian Academy of Sciences Budapest Hungary; ^4^ Department of Systematic Zoology and Ecology Faculty of Science Institute of Biology Eötvös Loránd University Budapest Hungary; ^5^ Plant Protection Institute Faculty of Agricultural and Environmental Sciences Szent István University Gödöllő Hungary

**Keywords:** wheat dwarf virus, WDV, monocot, *Hordeum vulgare*, barley, CRISPR/Cas9, resistance, *Psammotettix alienus*, leafhopper

*Wheat dwarf virus* (WDV) is an economically important, phloem‐limited, insect‐transmitted virus belonging to the *Geminiviridae* family (Tholt *et al*., 2018). WDV strains infect both wheat and barley causing severe yield losses and the natural resistance resources are limited (Nygren *et al*., 2015). Direct utilization of the CRISPR/Cas9 system to inhibit geminivirus replication has been described in model plants (Zaidi *et al*., 2016). Here, we show the direct antiviral utilization of the CRISPR/Ca9 system in an important crop plant, barley (*Hordeum vulgare* L. *cv*. Golden promise), to establish an effective WDV resistance.

To identify multiple target sites, we mapped the WDV genome for potential CRISPR/Cas9 target sequences encompassing the PAM motif. To create protection against multiple virus strains, the genomic sequence of two barley and two wheat WDV strains were used to identify potential sgRNA target sites located in conservative regions (Figure S1). Four target sites were selected which did not exhibit *in silico* predicted off‐target effects and attack different viral DNA segments (Table S1, Figure S2). The sgRNA_WDV1 shows complementarity to the overlapping region of the MP and CP coding sequence, sgRNA_WDV2 targets the Rep/RepA coding sequence at the N‐terminus of the proteins while sgRNA_WDV3 the LIR region, sgRNA_WDV4 targets genomic region encoding the C‐terminus of Rep (Figure 1a).

**Figure 1:**
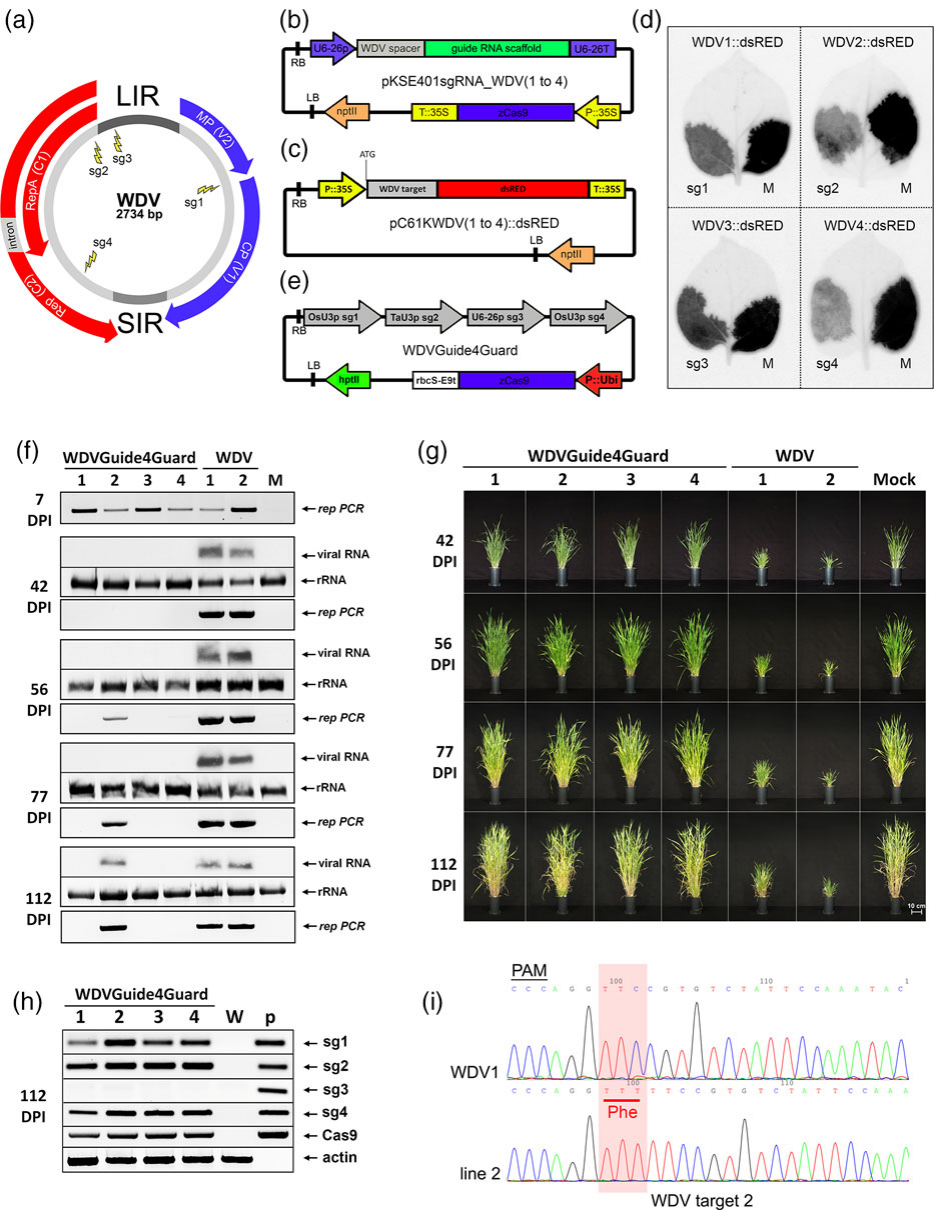
CRISPR/Cas9‐mediated WDV resistance in barley. (a) Schematic representation of the WDV genome with the four selected guide RNA target site positions (sg1, sg2, sg3, sg4). (b) Schematic representation of the dicotyledonous‐specific sgRNA and Cas9‐expressing binary vector. (c) Physical map of the sgRNA‐sensor vectors. The 24‐nt WDV target sequences with the PAM motif are fused individually to the dsRED‐encoding sequence in‐frame after the translation start codon (ATG). (d) The results of *Agrobacterium*‐mediated transient sensor tests in *Nicotiana benthamiana* leaves. Expression of the dsRED sensor constructs containing the four different WDV target sites (WDV1, WDV2, WDV3 or WDV4) co‐infiltrated with the corresponding sgRNAs (sg1, sg2, sg3 or sg4) or nonspecific guide RNA (M) at 3 days postinoculation. (e) Schematic representation of the WDVGuide4Guard binary vector with the four different WDV genome‐specific sgRNA sequences and the maize codon‐optimized *Cas9* gene controlled by the constitutive maize *Ubi1* promoter. (f) *Rep*‐specific PCR assays from inoculated leaves at 7 DPI (day postinfection) at the time of removal of WDV‐carrying leafhoppers (upper panel). Northern blot hybridization and *Rep*‐specific PCR assays from systemically infected leaves at the indicated time points (bottom panels). Relative gel loadings in the northern blot assay are indicated by ethidium bromide staining of the ribosomal RNAs (rRNA). (g) Phenotypic analysis of the WDV‐infected transgenic lines (WDVGuide4Guard1–4) in comparison with control‐infected (WDV1 and 2) and noninfected mock (M) wild‐type plants at different time points after infection (42, 56, 77, 112 DPI). Bar = 10 cm. (h) RT‐PCR analysis of the expressions of four sgRNAs (sg1, sg2, sg3, sg4) and the Cas9 RNA in transgenic lines (WDVGuide4Guard1–4) and wild‐type control (M) plant at 112 DPI. Relative RNA loadings are indicated by actin RT‐PCR as an internal control. p – WDVGuide4Guard plasmid DNA as PCR technical control. (i) Sequence analyses of sgRNA_WDV2 target site of PCR products originating from WDV genomes isolated from infected wild‐type (WDV1) and transgenic T0 plant (line 2). Red box indicates the inserted phenylalanine (Phe) coding TTT nucleotides in the mutant virus originated from line 2. PAM, protospacer adjacent motif.

An *Agrobacterium‐*mediated transient expression system was built to assess the biological activity of the sgRNA constructs on their target sequences. The individual sgRNA constructs were cloned into a binary vector [pKSE401; (Xing *et al*., 2014)] containing a 35S promoter‐driven Cas9 expression cassette (Figure 1b) and transformed into *Agrobacterium tumefaciens*. We generated transient *in vivo* sensor systems by introducing single 24‐nt long sgRNA target sequences containing the PAM region in‐frame after the start codon of the *dsRED* reporter gene. These sensor constructs were then cloned into binary vectors under the control of the 35S promoter (Figure 1c) and transformed into *A. tumefaciens*. The *Agrobacterium*‐mediated transient co‐transformation experiments were carried out by co‐infiltrating the dsRED sensor constructs with the particular sgRNAs; hence the inhibited activity of the dsRED reflects the activity of the sgRNAs (Figure 1d). We found that all the four sgRNAs inhibited the activity of the adequate sensor construct. Control agroinfiltration experiments confirmed the sequence‐specific actions of the tested WDV‐specific sgRNAs (Figure S3 and S4).

To produce transgenic plants a binary construct harbouring the four WDV‐specific sgRNAs (1‐4) under the control of three different monocotyledon‐specific small nuclear RNA promoters was constructed (WDVGuide4Guard) using the vector system described previously (Xing *et al*., 2014). This vector also expresses a codon‐optimized maize Cas9 under the control of the monocotyledon‐specific maize *Ubi1* promoter (Figure 1e). Barley plants (*cv*. Golden Promise) were used for *Agrobacterium*‐mediated transformation as described previously (Kis *et al*., 2016) and 20 transformants were collected from four independent calli. We selected four T0 lines, representatives of the four different calli, and checked the lines for the presence of the transgene casette by PCR analyses and sequencing (Figure S5). These transgenic plants were indistinguishable from the wild‐type barley plants indicating that the presence of the transgene cassette does not interfere with normal development. We used these T0 transgenic lines and control plantlets for challenge infection studies.

We produced WDV‐infected barley plants by *Agrobacterium*‐mediated delivery of an agroinfectious WDV clone, as described previously (Kis *et al*., 2016). These plants were used to feed the reared *Psammotettix alienus* (Dahlbom) leafhoppers to acquire WDV. Next, isolation chambers containing the WDV‐carrying leafhoppers were applied onto plants to mimic the natural infection process. The selected four transgenic lines and control plants were further grown in a climate chamber at 12–15 °C and challenge infected by WDV‐carrying leafhoppers at the 3–4 leaves stage. The infection processes of the transgenic and control barley plants were monitored by molecular techniques: PCR analysis, detecting the presence of viral genomic DNA while northern blot analyses of WDV‐specific Rep RNA quantitatively indicating active virus replication, and also by phenotypic observations (Figure 1f and g). In all the virus‐inoculated leaves, we could detect the presence of virus‐specific DNA by PCR at 7 days postinfection (DPI; Figure 1f). This observation indicates that the viral DNA has been successfully delivered by the vector insects. After 42 DPI, the control plants showed signs of dwarfing typical of WDV infection and the abundant accumulation of virus‐associated DNA and RNA products, while no signs of infection could be detected on the transgenic lines. However, at 56 DPI, although no visible disease symptoms were observed and the viral RNAs could not be detected by northern blot, the presence of viral DNA was confirmed by PCR in line 2. The other transgenic lines showed no virus presence. As the viral infection advances, the control plants showed severe viral disease symptoms and high level of virus accumulation (Figure 1f and g). WDVGuide4Guard_2 line exhibited effective virus tolerance since despite the accumulation of virus DNA and RNA products at 112 DPI and the plant showed normal phenotype and produced spikes similarly to the noninfected control plant. WDVGuide4Guard lines 1, 3 and 4 exhibited no viral symptoms, and the presence of the virus was detected neither by northern blot nor PCR analysis. These data indicated that these lines are fully resistant to the insect vector‐mediated WDV infection.

Next, we investigated the presence of different sgRNAs in the infected plants at 112 DPI. RT‐PCR analyses confirmed the expression of sgRNA_WDV1, sgRNA_WDV2 and sgRNA_WDV4, however, sgRNA_WDV3 did not accumulate in any of the lines (Figure 1h). The lack of proper accumulation of this sgRNA can be explained by the high T content of the spacer sequence of sgRNA_WDV3 which may result in transcriptional termination by RNA polymerase III (Hamada *et al*., 2000). We also found that there is no direct correlation between resistance and the level of Cas9 protein since the tolerant WDVGuide4Guard_2 line contained a high level of Cas9 while the resistant WDVGuide4Guard_1 produced less Cas9 (Figure S6). Next, we amplified the WDV strain present in WDVGuide4Guard_2 line by PCR to assess potential recombination events at the recognition sites of sgRNAs. The direct sequencing of the PCR products revealed that only the sgRNA_WDV2 worked, inducing a three‐nucleotide insertion in the WDV genome (Figure 1i). The sequence analyses of the three other sgRNA target sites recovered no changes (Figure S7). The absence of recombination events at the target sites of sgRNA_WDV1 and sgRNA_WDV4 suggests that the efficient activity of sgRNA_WDV2 can be responsible for the WDV resistance or tolerance. To test the heritability of the introduced trait of we collected seeds from a sibling plant of WDVGuide4Guard_2 (T0) line and investigated ten selected lines from T1 progeny plants. These healthy T1 plants were challenge infected by WDV (wild type strain) carrying leafhoppers similarly to the T0 plants. The virus delivery by leafhoppers was successful in all plants demonstrated by the presence of virus‐specific DNA by PCR at 7 DPI (Figure S8a). At 112 DPI, the control barley plants displayed the typical symptoms normally associated with WDV infections and high‐level accumulation of viral DNA and RNA products. In contrast, seven of the ten progeny lines showed no phenotypic signs of virus infection and no WDV‐derived products were detected by PCR or northern blot analyses. However, virus derived products accumulated in three progeny lines (2.1, 2.5 and 2.10; Figure S8a and b). Similarly to the T0 plants sgRNA_WDV3 did not accumulate in the progeny lines (Figure S8c) and Cas9 production was at high or average levels in lines which became WDV infected (Figure S9). The developing disease symptoms in infected T1 lines were different: 2.5. and 2.10 exhibited moderate and severe phenotypic alterations, respectively, while 2.1 showed no visible symptoms. Sequencing of the PCR products of mutant WDV strains revealed that 2.1 and 2.5 lines contained mixed sequence variants at the location of the sgRNA_WDV2 target site while a nucleotide substitution at this target site evolved a single recombinant WDV strain in the 2.10 (Figure S10). The presence of different, independently generated mutant WDV strains might responsible for the development of altered disease symptoms in T1 lines. Similarly to T0 plants the recombinant WDV strains do not display any mutations at the three other target sites, strongly suggesting that these sgRNAs are ineffective on the viral genome (Figure S10). These results indicate that the introduced trait is stably heritable mediating the expression of sgRNAs and providing WDV resistance or tolerance. The used oligonucleotides are listed in Table S2.

Our results demonstrate that in case of lacking natural resistance resources, the CRISPR/Cas9 system can be utilized to establish extremely efficient resistance in monocotyledonary plants to combat an economically important, insect vector‐transmitted, destructive DNA virus. However, the selection of potent sgRNAs and ensuring their proper expression are prerequisites of the optimal result. The co‐application of different alternative biotechnological techniques can provide a powerful solution for elaborating durable, long‐lasting, highly efficient broad‐spectrum resistance (Fuchs, 2017). The rapid technological evolution of genome editing techniques (Wu *et al*., 2018) and their adaptation to revolutionary new applications, such as direct targeting of viruses with RNA genomes (Aman *et al*., 2018; Zhang *et al*., 2018), will evolve this technology to one of the most powerful molecular biology tools enabling the fast introduction of efficient resistances against newly emerging pathogens.

## Conflict of interests

The authors declare no conflict of interests.

## Supporting information


**Figure S1** Positions of the 19 selected conservative target sequences with the PAM site on the alignment of two barley (AM747816.1, FM210034.1) and two wheat (FN806785, FN806786) infecting WDV genomes.
**Figure S2** The sequence details of the four selected target sites (virion‐sense orientation) with the spacer sequences of the sgRNAs.
**Figure S3.** T7 endonuclease assay for detecting DNA repair event in the dsRED sensor constructs. Red arrow indicates the T7 endonuclease cleavage products.
**Figure S4** Control experiment for potential unspecific activities of sgRNAs in *Agrobacterium*ߚmediated transient system in tobacco.
**Figure S5** PCR analysis of the T0 barley line (1‐4) with WDV_sg1D_F and Ubi1_det_5'_R, w – wild plant, p – WDVGuide4Guard plasmid, dv – distillated water.
**Figure S6** Cas9 western blot analysis of transgenic T0 barley lines (1‐4) and non‐infected wild type barley plant (M) at 112 days post infection (DPI).
**Figure S7** Sequence analysis of the resistance breaking WDV genome. WDV strains were isolated from infected wild‐type (WDV1) and the transgenic T0 plant (line 2) and sequence analyses were carried out at the four target sites of sgRNAs (WDV target 1‐4).
**Figure S8** Investigation of the T1 (line 2) progeny transgenic barley lines after insect‐mediated WDV infection.
**Figure S9** Cas9 western blot analysis of transgenic barley T1 (line 2) progeny plants and non‐infected wild type barley plant (Mock) at 112 DPI.
**Figure S10** Sequence analysis of the resistance breaking WDV genomes in T1 plants.
**Table S1** The potential off‐target effects of WDV specific sgRNAs on barley and wheat genomes based on Ensembl database (http://plants.ensembl.org) BLAST.
**Table S2** List of oligos used in this work.
