# Supplementary material for: Creating highly efficient resistance against wheat dwarf virus in barley by employing CRISPR/Cas9 system
Source: Plant Biotechnol J. 2019 Feb 5;17(6):1004–6. doi: 10.1111/pbi.13077 (PMC6523583; doi:10.1111/pbi.13077)
Supplement: Supplementary file 1 — Figure S1 Positions of the 19 selected conservative target sequences with the PAM site on the alignment of two barley (AM747816.1, FM210034.1) and two wheat (FN806785, FN806786) infecting WDV genomes. Figure S2 The sequence details of the four selected target sites (virion‐sense orientation) with the spacer sequences of the sgRNAs. Figure S3. T7 endonuclease assay for detecting DNA repair event in the dsRED sensor constructs. Red arrow indicates the T7 endonuclease cleavage products. Figure S4 Control experiment for potential unspecific activities of sgRNAs in Agrobacteriumߚmediated transient system in tobacco. Figure S5 PCR analysis of the T0 barley line (1‐4) with WDV_sg1D_F and Ubi1_det_5'_R, w – wild plant, p – WDVGuide4Guard plasmid, dv – distillated water. Figure S6 Cas9 western blot analysis of transgenic T0 barley lines (1‐4) and non‐infected wild type barley plant (M) at 112 days post infection (DPI). Figure S7 Sequence analysis of the resistance breaking WDV genome. WDV strains were isolated from infected wild‐type (WDV1) and the transgenic T0 plant (line 2) and sequence analyses were carried out at the four target sites of sgRNAs (WDV target 1‐4). Figure S8 Investigation of the T1 (line 2) progeny transgenic barley lines after insect‐mediated WDV infection. Figure S9 Cas9 western blot analysis of transgenic barley T1 (line 2) progeny plants and non‐infected wild type barley plant (Mock) at 112 DPI. Figure S10 Sequence analysis of the resistance breaking WDV genomes in T1 plants. Table S1 The potential off‐target effects of WDV specific sgRNAs on barley and wheat genomes based on Ensembl database (http://plants.ensembl.org) BLAST. Table S2 List of oligos used in this work. [file PBI-17-1004-s001.docx]

## Supplementary Figures and Tables


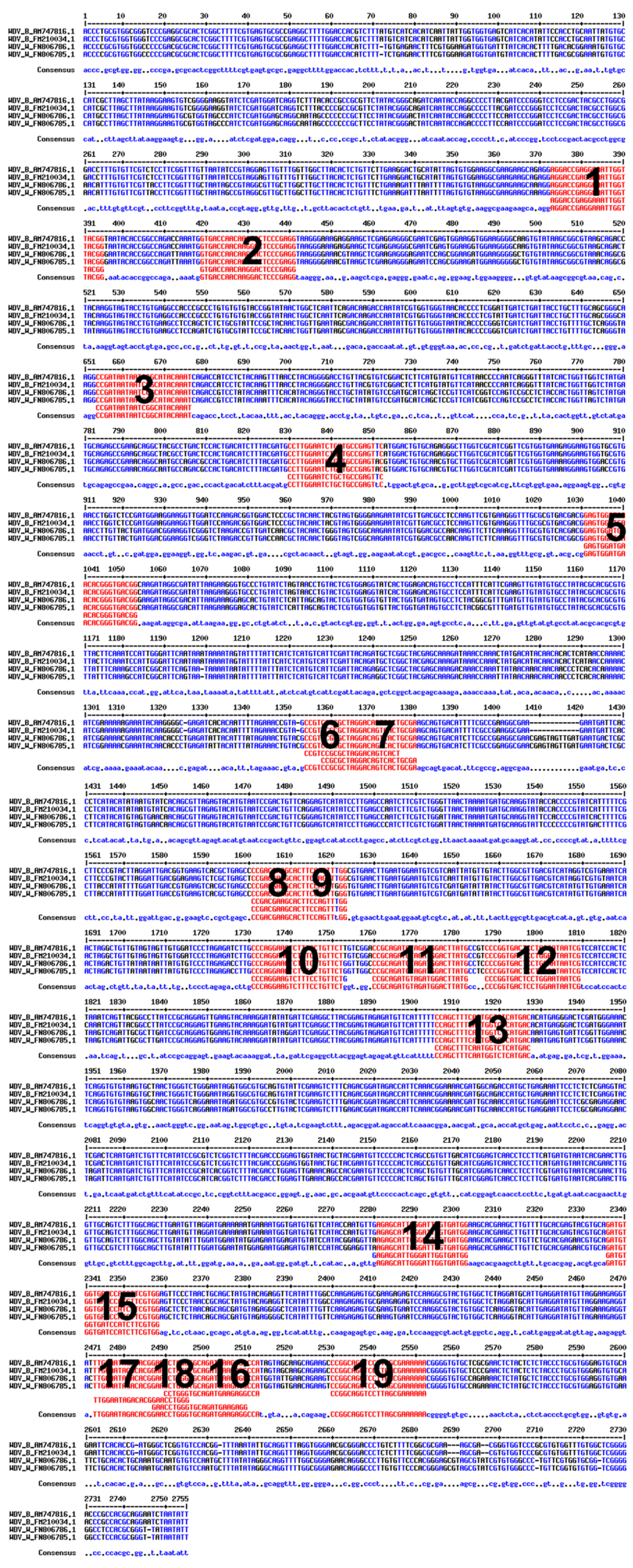


**Figure S1** Positions of the 19 selected conservative target sequences with the PAM site on the alignment of two barley (AM747816.1, FM210034.1) and two wheat (FN806785, FN806786) infecting WDV genomes.


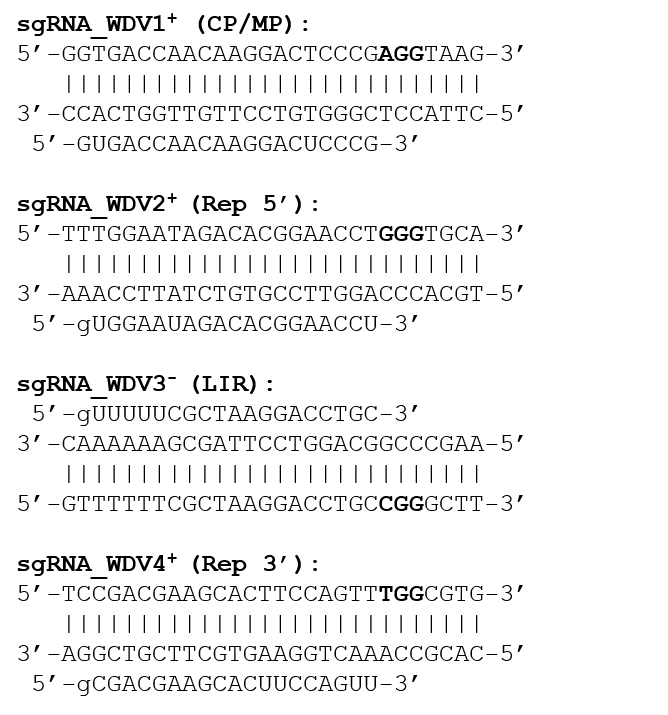


**Figure S2** The sequence details of the four selected target sites (virion-sense orientation) with the spacer sequences of the sgRNAs. Bold letters indicate the PAM (NGG; Protospacer Adjacent Motif) site.

**
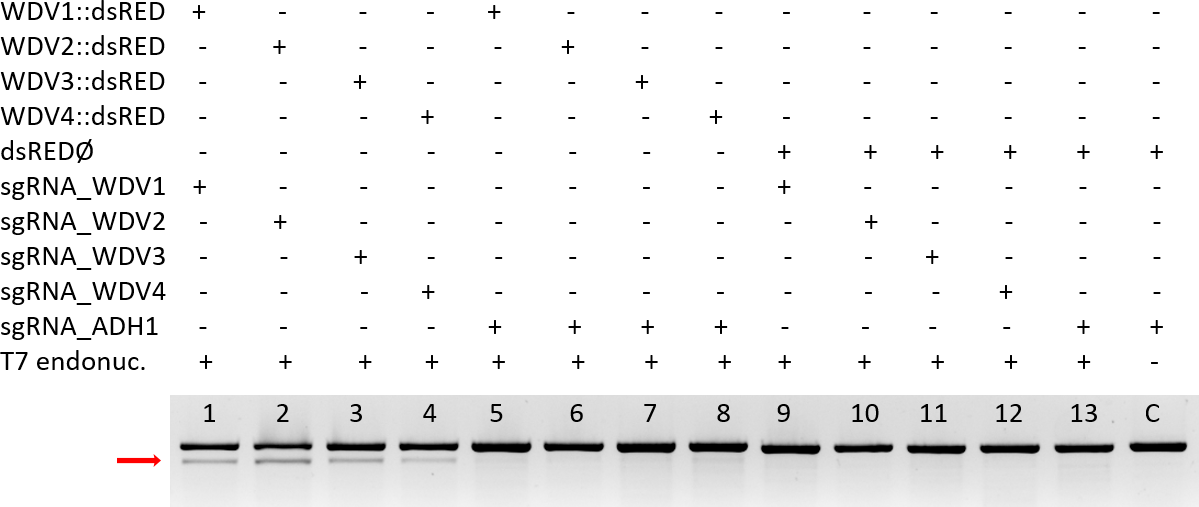
**

**Figure S3** T7 endonuclease assay for detecting DNA repair event in the dsRED sensor constructs. Red arrow indicates the T7 endonuclease cleavage products.


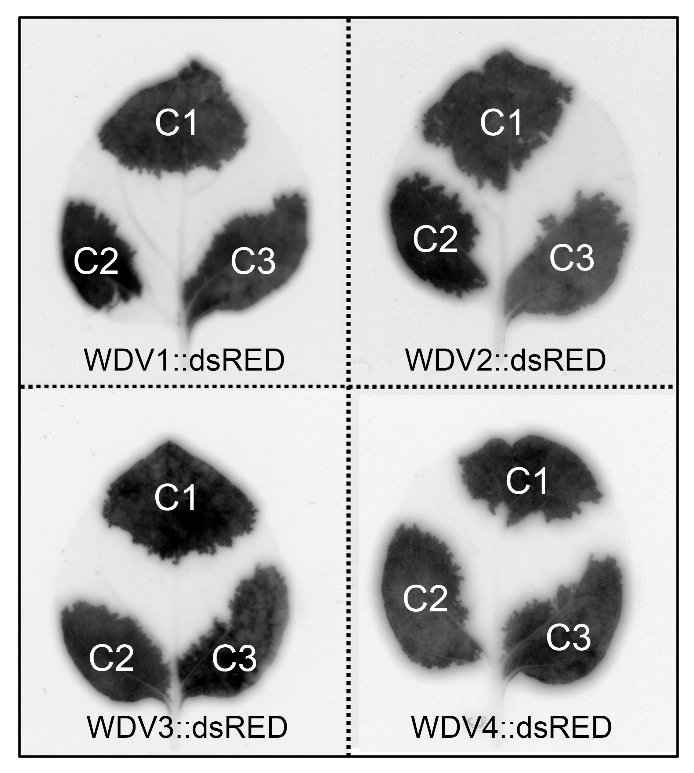


**Figure S4** Control experiment for potential unspecific activities of sgRNAs in *Agrobacterium*-mediated transient system in tobacco. C1 – pKSE401ADH1sg (non-specific guide RNA) co-infiltrated with the pC61KdsRED vector; C2 - pKSE401ADH1sg co-infiltrated with the corresponding sgRNA sensor constructs (pC61KWDV(1 to 4)::dsRED); C3 - pC61KdsRED co-infiltrated with the corresponding sgRNA constructs (pKSE401sgRNA_WDV(1 to 4)).


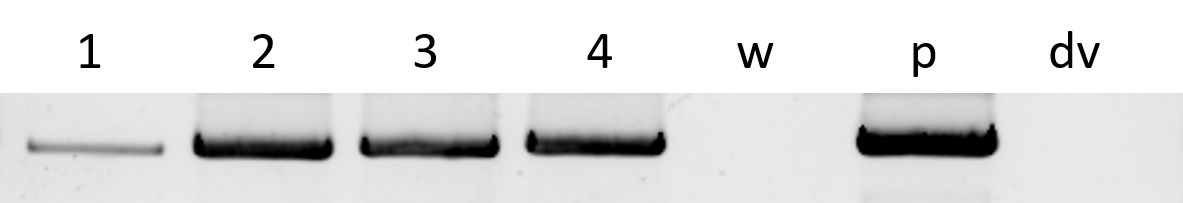


**Figure S5** PCR analysis of the T0 barley line (1-4) with WDV_sg1D_F and Ubi1_det_5’_R, w – wild plant, p – WDVGuide4Guard plasmid, dv – distillated water. The PCR products have been sequenced and the sequence analyses confirmed the presence of the transgene in the investigated lines (data not shown).

**
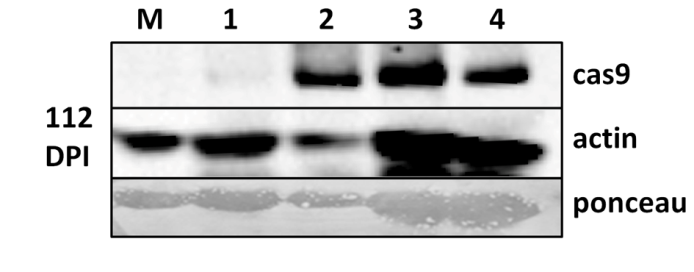
**

**Figure S6** Cas9 western blot analysis of transgenic T0 barley lines (1-4) and non-infected wild type barley plant (M) at 112 days post infection (DPI). Relative protein loadings are indicated by actin western blot and ponceau staining.


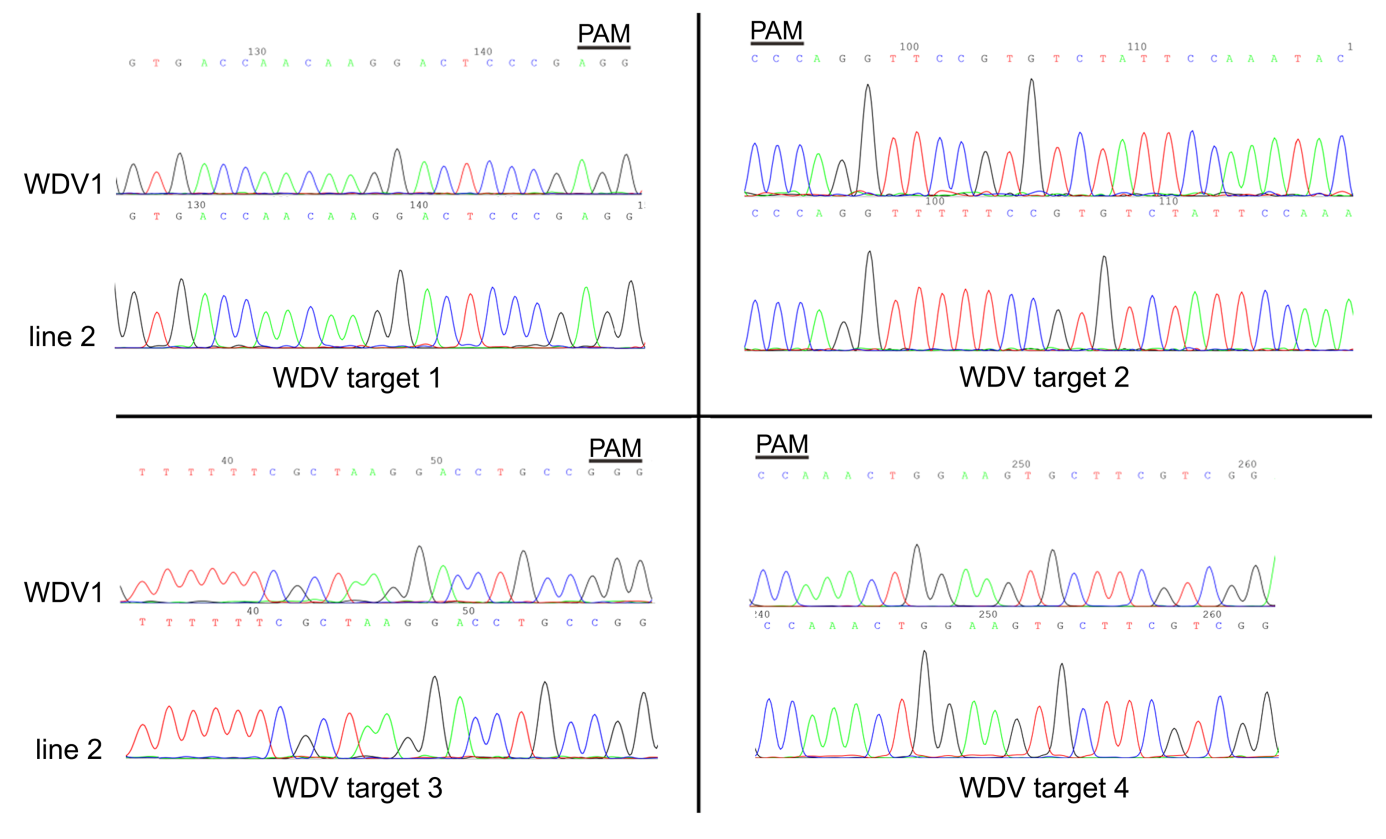


**Figure S7** Sequence analysis of the resistance breaking WDV genome. WDV strains were isolated from infected wild-type (WDV1) and the transgenic T0 plant (line 2) and sequence analyses were carried out at the four target sites of sgRNAs (WDV target 1-4). PAM - Protospacer Adjacent Motif.


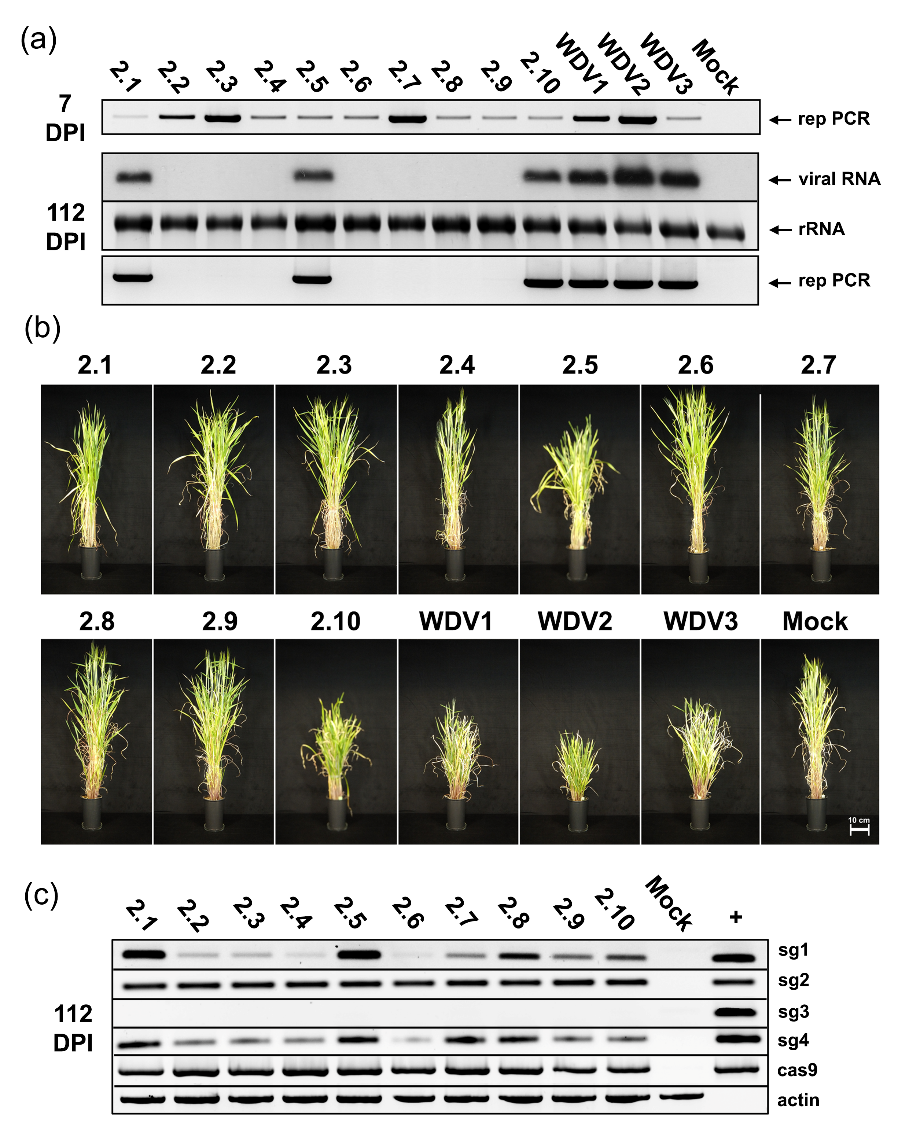


**Figure S8** Investigation of the T1 (line 2) progeny transgenic barley lines after insect-mediated WDV infection. (a) northern blot hybridization and *Rep* specific PCR assays from inoculated leaves at 7 DPI after removal of the WDV-carrying leafhoppers and from systemically infected leaves at 112 DPI. Relative gel loadings in the northern blot assay are indicated by ethidium bromide staining of the ribosomal RNAs (rRNA). (b) Phenotypic analysis of the WDV-infected T1 progeny transgenic lines (WDVGuide4Guard(1 to 4)) in comparison with control-infected (WDV1 to 3) and non-infected mock (M) plants at 112 (DPI)). Bar = 10 cm**.** (c) RT-PCR analysis of the transgenic T1 plants (line2) progenies and wild-type control (M) plants for expression of sgRNAs (sg1, sg2, sg3, sg4) and the Cas9 RNA at 112 DPI. Relative RNA loadings are indicated by actin RT-PCR as an internal control. p – WDVGuide4Guard plasmid DNA as PCR technical control.


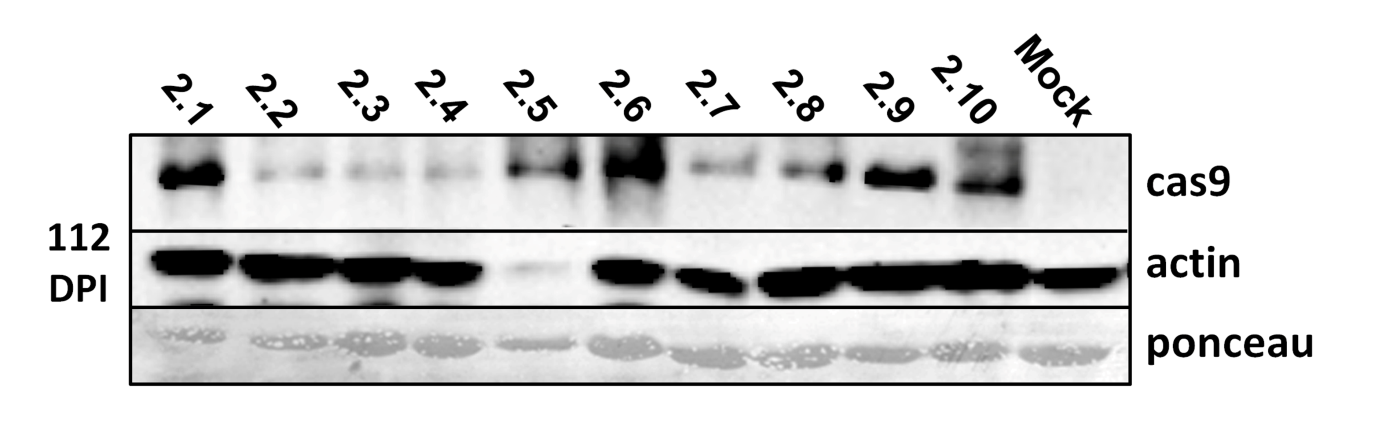


**Figure S9** Cas9 western blot analysis of transgenic barley T1 (line 2) progeny plants and non-infected wild type barley plant (Mock) at 112 DPI. Relative protein loadings are indicated by actin western blot and ponceau staining.

**
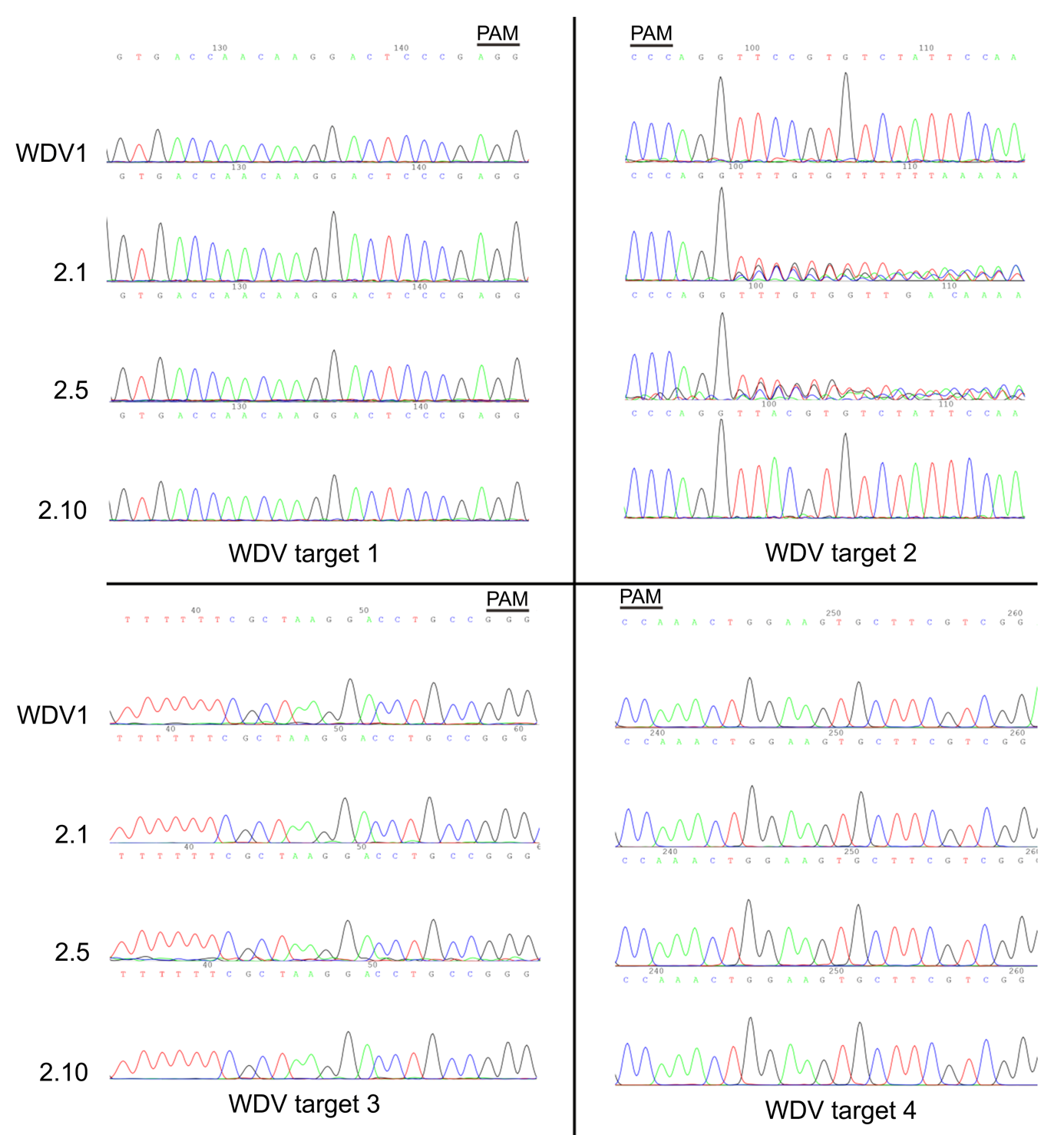
**

**Figure S10** Sequence analysis of the resistance breaking WDV genomes in T1 plants. WDV strains were isolated from infected wild-type (WDV1) and the transgenic T1 plants (2.1, 2.5 and 2.10) and sequence analyses were carried out at the four target sites of sgRNAs (WDV target 1-4). PAM - Protospacer Adjacent Motif.

| **no.** | **Target sequences** | **Target gene** | **G/C ratio (%)** | **OFF-target in barley genome** | **OFF-target in wheat genome** |
| --- | --- | --- | --- | --- | --- |
|  |  |  |  |  |  |
| 01 | AGGACCGAGGAAATTGGTTA CGG | MP | 45 | 0 | 0 |
| **02** | **GTGACCAACAAGGACTCCCG AGG** | **CP/MP** | **60** | **0** | **0** |
| 03 | CCG ATAATAATCGGCATACAAAT | CP | 25 | 0 | 0 |
| 04 | CCT TGGAATCTGCTGCCGAGTTC | CP | 55 | 0 | 1 |
| 05 | GAGTGGATGAACACGGGTGA CGG | CP | 55 | 0 | 0 |
| 06 | CCG TCCGCGCTAGGACAGTCACT | SIR | 60 | 0 | 2 |
| 07 | CCG CGCTAGGACAGTCACTGCGA | SIR | 60 | 0 | 0 |
| 08 | CCG ACGAAGCACTTCCAGTTTGG | REP | 50 | 0 | 0 |
| **09** | **CCGACGAAGCACTTCCAGTT TGG** | **REP** | **55** | **0** | **0** |
| 10 | CCC AGGAAGTCTTTCCTGTTCTG | REP | 45 | 0 | 0 |
| 11 | CCG CAGATGTAGATGGACTTATG | REP | 40 | 1 | 4 |
| 12 | CCC GGTGACTCCTGGAATAATCG | REP | 50 | 0 | 1 |
| 13 | CCA GCTTTCAATGGTCTCATGAC | REP | 45 | 0 | 0 |
| 14 | GAGAGCATTGGGATTGGTGA TGG | REP | 50 | 1 | 2 |
| 15 | GATGTGGTGATCCATCTTCG TGG | REP | 50 | 6 | 39 |
| 16 | CCT GGGTGCAGATGAAGAGGCCA | REP | 60 | 0 | 1 |
| **17** | **TTGGAATAGACACGGAACCT GGG** | **REP** | **45** | **0** | **0** |
| 18 | GAACCTGGGTGCAGATGAAG AGG | REP | 55 | 1 | 12 |
| **19** | **CCG GCAGGTCCTTAGCGAAAAAA** | **LIR** | **45** | **0** | **0** |

**Table S1** The potential off-target effects of WDV specific sgRNAs on barley and wheat genomes based on Ensembl database (http://plants.ensembl.org) BLAST. Bold lines indicate selected sgRNA target sites (no. 2=sgRNA_WDV1, no. 09=sgRNA_WDV4, no. 17=sgRNA_WDV2, no. 19=sgRNA_WDV3).

| **Primers for guide RNA and sensor constructions** | |
| --- | --- |
|  | |
| For pKSE401 (dicot-specific) CRISPR constructions | |
| **WDV_sg1D_F** | ATTGTGACCAACAAGGACTCCCG |
| **WDV_sg1D_R** | AAACCGGGAGTCCTTGTTGGTCA |
| **WDV_sg2D_F** | ATTGTGGAATAGACACGGAACCT |
| **WDV_sg2D_R** | AAACAGGTTCCGTGTCTATTCCA |
| **WDV_sg3D_F** | ATTGTTTTTCGCTAAGGACCTGC |
| **WDV_sg3D_R** | AAACGCAGGTCCTTAGCGAAAAA |
| **WDV_sg4D_F** | ATTGCGACGAAGCACTTCCAGTT |
| **WDV_sg4D_R** | AAACAACTGGAAGTGCTTCGTCG |
| **ADH1_sg_F** | ATTGGTATCTTCGGCCATGAAGC |
| **ADH1_sg_R** | AAACGCTTCATGGCCGAAGATAC |
| For pHUE411 (monocot-specific) CRISPR construction | |
| **WDV_sg1F** | ATATAT**GGTCTC**TGGCGTGACCAACAAGGACTCCCGGTTTTAGAGCTAGAAATAGC |
| **MT0-BsR2** | ATTATT**GGTCTC**TGCTTCTTGGTGCCGC |
| **WDV_sg2F** | ATATAT**GGTCTC**TAAGCGTGGAATAGACACGGAACCTGTTTTAGAGCTAGAAATAGC |
| **MT0-BsR3** | ATTATT**GGTCTC**TCAATCACTACTTCGACTCTAGC |
| **WDV_sg3F** | ATATAT**GGTCTC**TATTGTTTTTCGCTAAGGACCTGCGTTTTAGAGCTAGAAATAGC |
| **WDV_sg4R** | ATTATT**GGTCTC**TAAACAACTGGAAGTGCTTCGTCGCGCCACGGATCATCTGCACAACT |
| For pC61KdsRED construction | |
| **dsRED_KpnI_F** | tta**GGTACC**ATGGCGCGCTCCTCCAAGAAC |
| **dsRED_XbaI_R** | gtc**TCTAGA**CCTACAGGAACAGGTGGTGGC |
| For dsRED sensor constructions | |
| **WDVsg1::dsRED_KpnI_F** | tta**GGTACC**atgGTGACCAACAAGGACTCCCGAGGTgcgcgctcctccaaga |
| **WDVsg2::dsRED_KpnI_F** | tta**GGTACC**atgACCCAGGTTCCGTGTCTATTCCAAgcgcgctcctccaaga |
| **WDVsg3::dsRED_KpnI_F** | tta**GGTACC**atgCCCGGCAGGTCCTTAGCGAAAAAAgcgcgctcctccaaga |
| **WDVsg4::dsRED_KpnI_F** | tta**GGTACC**atgCCGACGAAGCACTTCCAGTTTGGCgcgcgctcctccaaga |
|  | |
| **For T7 endonuclease assay** | |
| **35SP_F** | TCCCACTATCCTTCGCAAGACCC |
| **dsRED_XbaI_R** | gtc**TCTAGA**CCTACAGGAACAGGTGGTGGC |
| **For guide guide RNA cassettes detecting** | |
| **WDV_sg1D_F** | ATTGTGACCAACAAGGACTCCCG |
| **Ubi1_det_5’_R** | CTGCACTGCAGGCATGCAAGC |
| **To detect guide RNAs with RT-PCR** | |
| **WDV_sg1_det_F** | GTGACCAACAAGGACTCCCG |
| **WDV_sg2_det_F** | GTGGAATAGACACGGAACCT |
| **WDV_sg3_det_F** | GTTTTTCGCTAAGGACCTGC |
| **WDV_sg4_det_F** | GCGACGAAGCACTTCCAGTT |
| **gRNA_det_R** | GCACCGACTCGGTGCCACTTTTTC |
| **For WDV sequencing** | |
| **WDV_F1** | GGATTGGTGATGGAAGCACG |
| **WDV_R1** | CGTCACCCGTGTTCATCCACTCC |
| **WDV_F2** | GACTCCTGGAATAATCGTCCA |
| **WDV_R2** | CTGCTTTGCAGCGGGCAAGGCCG |
| **WDV_sg1_seq** | ATGCCGATTATTATCGGCC |
| **WDV_sg2_sg3_seq** | ATCGGTGTGAATTCTGCACAC |
| **WDV_sg4_seq** | GACTCCTGGAATAATCGTCCA |
| **For WDV detection** | |
| **WDVrepDetF** | CGCCTTGGACTCTCTTCGCAC |
| **WDVrepDetR** | GACGGATAGACCATTCAAACG |

**Table S2** List of oligos used in this work. Bold letters indicate restriction enzyme sites. The guide RNA sequences are highlighted in yellow.

## Supplementary Methods

*Plant materials and insect maintenance*

*N.* *benthamiana* plants grown in soil in growing chamber (Versatile Environmental Test Chambers; Sanyo, Tokyo Japan) under a 14-hour light (50 µE m^-2^s^-1^) and 10-hour dark cycles at 23 °C were used for agroinfiltration experiments. *Hordeum vulgare* *cv.* Golden promise plants were grown under 15 °C daytime and 12 °C night temperatures with 16-hour light (50 µE m^-2^s^-1^) and 8-hour dark periods to obtain material for *Agrobacterium-*mediated genetic transformation. Transgenic barley plants were kept under the same conditions. A pooled population of *Psammotettix alienus* (Dahlbom), originally collected from a barley field was maintained for several generations on barley (*H. vulgare* cv. Conchita) kept in growing chambers under a 14-hour light (50 µE m^-2^s^-1^) and 10-hour dark cycles at 23 °C on 30-40% relative humidity. Leafhoppers were kept on potted plants covered with a fine mesh (0.1 mm mesh size).

*Selection of CRISPR/Cas9 target sites*

Based on sequence analysis of two barley (AM747816, FM210034) and two wheat (FN806785, FN806786) specific WDV strains, 19 conservative Cas9 target sites were determined (Fig. S1). Guide RNAs specific to these target sites were analyzed for off-target effects: the sequences of the target sites were searched against the barley (Hv_IBSC_PGSB_v2) and wheat (TGACv1) genomes by BLAST (http://plants.ensembl.org). Sequences with PAM motif and upstream 12 base pairs of perfect complementarity with barley or wheat genomes were considered to have off-target effect (Table S1). Four potential guide RNAs with no off-target effects on the barley and wheat genomes were selected targeting different segments on the WDV DNA genome; sgRNA_WDV1: MP 3’ end and CP 5’ end (overlapping ORFs); sgRNA_WDV2: Rep/RepA 5’ end; sgRNA_WDV3: LIR; sgRNA_WDV4: Rep 3’ end.

*Plasmid constructs*

The dicot and monocot-specific CRISPR/Cas9 transformation vectors were constructed by BsaI-based Golden Gate cloning system using the pKSE401 and the pHUE411 plasmids, respectively, according to the authors’ instructions ([Xing et al., 2014](#_ENREF_3)). As a results, we got the one-sgRNA-expressing pKSE401sgRNA_WDV(1 to 4) vectors and the four-sgRNA-expressing monocot-specific construction WDVGuide4Guard. We used an *Arabidopsis thaliana* *ALCOHOL DEHYDROGENASE 1* gene (*ADH1*, AT1G77120) specific sgRNA as negative control. The pC61KWDV(1 to 4)::dsRED sensor constructions were created from a PCR-amplified dsRED fragments containing of each sgRNA’s target site in frame with ATG start codon and the pC61K plasmid ([Kis et al., 2016](#_ENREF_1)). The DNA primers of WDVsg(1 to 4)::dsRED_KpnI_F and and dsRED_XbaI_R were used to PCR amplify the target site encompassing dsRED fragments from pKGW-R vector ([Smit et al., 2005](#_ENREF_2)) and cloned into 35S cassette of pC61K plasmid. For control experiments, pC61KdsRED vector was constructed with dsRED_KpnI_F and dsRED_XbaI_R DNA primers. Table S2 contains the list of oligonucleotides used for cloning.

*Agrobacterium tumefaciens infiltration*

Agroinfiltration procedure was performed as described previously ([Kis et al., 2016](#_ENREF_1)). The optical density of each AGL-1 *Agrobacterium* strains hosting binary plasmids pC61KdsRED or variants of pC61KWDV(1 to 4)::dsRED were adjusted to OD_600_ = 1 and mixed in a 1:1 ratio with *Agrobacterium* suspension containing the pKSE401sgRNA_WDV(1 to 4) or non-specific guide RNA vector (pKSE401ADH1sg), then infiltrated into the leaves of young *N. benthamiana* plants. Three days after the infiltrations, the leaves were analyzed for dsRED signals using ChemiDoc™ MP Imaging System (Bio-Rad) and Image Lab (ver. 5.2) software (DyLight549 protocol). The experiments were replicated three times independently.

*Barley transformation and WDV infection*

200 immature barley (cv. Golden promise) embryos were transformed with *A. tumefaciens* AGL-1 harboring the WDVGuide4Guard construct as described previously ([Kis et al., 2016](#_ENREF_1)). 20 transformed plants were collected from 4 independent calli. The presence of the transgene was detected by PCR using the WDV_sg1D_F - Ubi1_det_5’_R primer pairs (Table S2), specific for sgRNA_WDV1 sequence and the maize *ubiquitin* (*Ubi1*) promoter amplifying a 2890 bp PCR fragment. Different individual plants of the four lines were selected for the WDV infection tests. The infection was based on the protocol we described earlier ([Kis et al., 2016](#_ENREF_1)) using *Agrobacterium*- and insect (*P. alienus*) vector-mediated infection. Briefly, artificially WDV-infected barley plants were used to feed the virus-free adult *P. alienus* stock-population in micro isolator chamber. After a week-long feeding, the virus carrying leafhoppers were moved to transgenic plants using two micro isolator chambers with 3-3 insects per plant for a week.

*RNA isolation and northern blotting*

Total RNA was extracted from agroinfiltrated and virus-infected plants at different time points using TRI® Reagent RNA Isolation Reagent (Sigma-Aldrich). For northern blot analyses of viral RNAs, 5 µg total RNA was separated on 1.2% formaldehyde agarose gels and blotted to Nytran NX membrane (Schleicher & Schuell, Germany). For WDV RNA detection, a 413 bp long WDV-specific PCR product (with WDVrepDetF and WDVrepDetR primers) was radioactively labelled using random priming probes by the Decalabel DNA labelling kit (Fermentas), as described previously ([Kis et al., 2016](#_ENREF_1)).

*PCR and RT-PCR analysis*

PCR reaction for detecting WDV replicase gene was performed as described previously ([Kis et al., 2016](#_ENREF_1)). To sequence the target sites on the WDV genomes, two fragments were amplified. The first was made using WDV_F1 and WDV_R1 primers producing a fragment containing sgRNA_WDV1, sgRNA_WDV2, sgRNA_WDV3 target sites. This has been sequenced using WDV_sg1_seq and WDV_sg2_sg3_seq primers. The sgRNA_WDV4 target site was amplified with WDV_F2 and WDV_R2 primers and sequenced with the WDV_sg4_seq primer. For RT-PCR, 5 μg of the DNase-treated (DNase I, NEB) RNA were used following the manufacturer’s instructions. After the DNase treatment, RNA was precipitated with absolute ethanol, then dried and resuspended in milliQ water. The cDNA was synthetized using the RevertAid Reverse Transcriptase kit (Thermo Fischer) following the manufacturer’s instructions with minor modifications: equal volumes of random hexamer and gRNA_det_R (designed to the guide RNS scaffold 3'-end) oligonucleotides were used for the first-strand synthesis. To detect the guide RNAs we used the gRNA_det_R oligonucleotide with the corresponding guide RNA‘s detecting forward primer (WDV_sg( 1 to 4)_det_F). PCR primers are listed in Table S2.

*T7 endonuclease assay*

To detect DNA repair events following Cas9 mediated cleavage on the infiltrated dsRED sensor constructs, we used T7 endonuclease I (NEB). Three days after infiltrations DNA was extracted from the infiltrated leaves using Qiagen DNeasy Plant Mini Kit according to the manufacturer’s instructions. We used 50 ng DNA extract for PCR template to produce an 838 bp long PCR fragment using the 35SP_F - dsRED_XbaI_R oligo pairs (Table S2). 5 µl unpurified PCR fragment from the reaction was used in 10 µl T7 endonuclease reaction, containing 1 U enzyme, and incubated for 30 minutes at 37 ˚C.

*Western blot analysis*

We homogenized the barley and *N. benthamiana* leaf samples in liquid nitrogen. 100 mg of tissue powder was mixed with 400 μl extraction buffer (10 mM Tris-HCl, 1 mM EDTA, 150 mM NaCl, 10 V/V% glicerol, 0,5 V/V% Nonidet P-40, 5 mM NaF, 1 mM DTT, 100 mM PMSF, 200 mM Na_3_VO_4_). The sediment was removed by centrifugation (4°C, 15 minutes, 1500 rpm). The supernatant was mixed with equal volume of 2× Laemmli buffer, and denatured at 72°C for 10 minutes. Protein samples were resolved on an 8% SDS-polyacrylamide gel and transferred to a PVDF Blotting Membrane (Amersham™ Hybond™) with overnight electro-blotting and subjected to western blot analysis. The membrane was blocked with 5% non-fat milk powder in PBS containing 0.1% Tween 20 (PBST) for 60 minutes. We probed the membrane with anti-Cas9 (Agrisera) in PBST containing 1% non-fat milk powder at room temperature for 160 minutes with agitation. After washing the membrane in PBST, we added the secondary goat anti-rabbit IgG (Agrisera). The membrane was incubated at room temperature for 90 minutes with agitation. The signals were visualized by chemiluminescence (ClarityTM Western ECL substrate; BIO-RAD) according to the manufacturer’s instructions. We stained the membrane with Ponceau reagent to check the protein loading.

**References**

Kis, A., Tholt, G., Ivanics, M., Varallyay, E., Jenes, B. and Havelda, Z. (2016) Polycistronic artificial miRNA-mediated resistance to Wheat dwarf virus in barley is highly efficient at low temperature. *Mol Plant Pathol* **17**, 427-437.

Smit, P., Raedts, J., Portyanko, V., Debelle, F., Gough, C., Bisseling, T. and Geurts, R. (2005) NSP1 of the GRAS protein family is essential for rhizobial Nod factor-induced transcription. *Science* **308**, 1789-1791.

Xing, H.L., Dong, L., Wang, Z.P., Zhang, H.Y., Han, C.Y., Liu, B., Wang, X.C. and Chen, Q.J. (2014) A CRISPR/Cas9 toolkit for multiplex genome editing in plants. *Bmc Plant Biol* **14**.
